# A dataset of definitive endoderm and hepatocyte differentiations from human induced pluripotent stem cells

**DOI:** 10.1038/s41597-023-02001-9

**Published:** 2023-02-14

**Authors:** Yuki Tanaka, Erina Furuhata, Shiori Maeda, Mami Kishima, Harukazu Suzuki, Takahiro Suzuki

**Affiliations:** 1 RIKEN Center for Integrative Medical Sciences, Yokohama, Kanagawa 230-0045 Japan; 2 Graduate School of Medical Life Science, Yokohama City University, Yokohama, Kanagawa 230-0045 Japan

**Keywords:** DNA methylation, Transcriptomics

## Abstract

Hepatocytes are a major parenchymal cell type in the liver and play an essential role in liver function. Hepatocyte-like cells can be differentiated *in vitro* from induced pluripotent stem cells (iPSCs) via definitive endoderm (DE)-like cells and hepatoblast-like cells. Here, we explored the *in vitro* differentiation time-course of hepatocyte-like cells. We performed methylome and transcriptome analyses for hepatocyte-like cell differentiation. We also analyzed DE-like cell differentiation by methylome, transcriptome, chromatin accessibility, and GATA6 binding profiles, using finer time-course samples. In this manuscript, we provide a detailed description of the dataset and the technical validations. Our data may be valuable for the analysis of the molecular mechanisms underlying hepatocyte and DE differentiations.

## Background & Summary

Hepatocytes compose approximately 70–85% of the liver mass and are crucial for the normal functioning of the liver. They are involved in activities such as detoxification, glycolytic and urea metabolism, control of blood cholesterol levels, and production of bile and hormones. Hepatocyte deficiencies such as hepatitis, non-alcoholic steatohepatitis (NASH), cirrhosis, and liver cancer result in severe health problems.

There is a great need for hepatocytes in medical and pharmaceutical applications. Because the liver is a central organ for foreign compound metabolism, hepatocytes are sensitive to drug toxicity. Therefore, hepatocytes isolated from the liver are also used to analyze pharmacokinetics and hepatotoxicity *ex vivo*. In addition, liver transplantation is an effective approach to the treatment of hepatic disorders, particularly end-stage liver disease. *Ex vivo* generation of the hepatocyte/liver is a promising alternative to the use of *in vivo* liver for such purposes.

During embryogenesis, hepatocytes are sequentially differentiated from pluripotent stem cells (PSCs) in the inner cell mass, definitive endoderm (DE) cells, and hepatoblasts. Many protocols for *in vitro* hepatic differentiation from PSCs, such as embryonic stem cells and induced pluripotent stem cells (iPSCs), have been described with different efficiencies and functionalities^1–8^. One of the earliest protocols, which is a standardized serum- and feeder-free differentiation protocol, showed highly efficient near-homogenous hepatocytic differentiation from a large panel of hPSC lines^8^. This protocol essentially mimics *in vivo* differentiation in three steps. First, PSCs differentiate into DE-like cells. Second, DE-like cells are committed to hepatoblast-like cells via the ventral foregut-like cells. Finally, the hepatoblast-like cells differentiated into fetal-like hepatocyte-like and hepatocyte-like cells. Because *in vitro* differentiated hepatocyte-like cells express key enzymes for detoxification, they are expected to be used in drug metabolism models.

During this process, precise successive alteration of gene expression profiles is essential for hepatic differentiation, which is governed by several key transcription factors. For example, GATA6 is a pivotal transcription factor for DE commitment and is, therefore, essential for liver development^9^.

In addition to transcription factors, gene expression is regulated by epigenome and chromatin levels. Importantly, the epigenome and chromatin structure are also controlled by proteins, indicating a complex regulatory mechanism among these factors.

In the present study, we explored changes in gene expression and DNA methylation using cap analysis gene expression (CAGE) and Infinium MethylationEPIC human methylation beadchips with a robust, standardized serum- and feeder-free hepatocyte differentiation protocol^8^. Furthermore, we analyzed gene expression, DNA methylation, chromatin accessibility, and GATA6 binding profile in the time range of DE-like cell commitment. Using this dataset, we previously investigated the regulatory relationship between transcription factors, epigenome, and chromatin structure^10^. In addition, our comprehensive time-course omics dataset can be reused for a detailed analysis of the molecular mechanisms underlying hepatocyte-like cell differentiation. Notably, taking advantage of CAGE, which provides highly quantitative transcriptional start sites and their activity, analysis of enhancers, lncRNAs, and alternative promoters is also available.

## Methods

### Study design

**Fig. 1:**
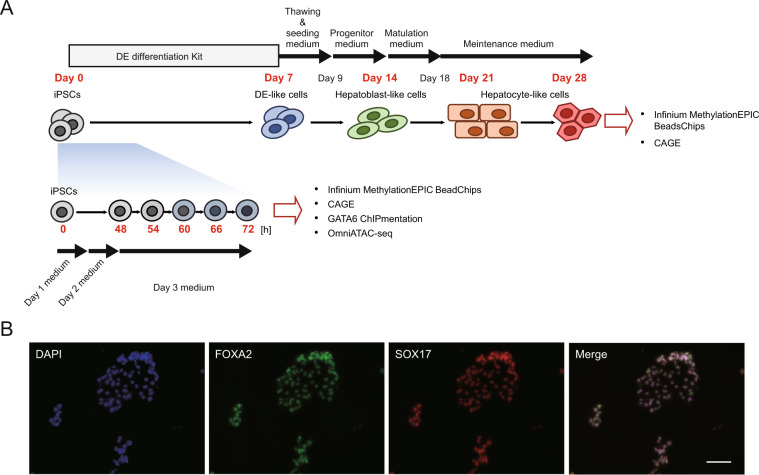
Data generation. (**A**) Time-course samples of *in vitro* hepatocyte differentiation were obtained at 0, 7, 14, 21, and 28 days after initiation of the differentiation. The obtained samples were subjected to Infinium MethylationEPIC beadschips and CAGE. Time-course samples of *in vitro* DE differentiation were obtained 0, 48, 54, 60, 66, and 72 hours (h) after initiation of the differentiation. The obtained samples were subjected to Infinium MethylationEPIC beadschips, CAGE, GATA6 ChIPmentation, and OmniATAC-seq. The time points when samples were collected are shown as bold-red characters. The culture condition of hepatocyte and DE differentiation are shown at the upper and lower of each schematic, respectively. (**B**) Immunocytochemistry for FOXA2 and SOX17 in Day 7 DE-like cells. The scale bar is 100 μm.

Figure 1A illustrates the dataset acquired in this study. We obtained two *in vitro* differentiation time-course datasets: iPSCs to hepatocyte-like cells and iPSCs to DE-like cells. The hepatic differentiation was performed using the Cellartis® Hepatocyte Differentiation Kit (Takara Bio Inc., Shiga, Japan). We collected the samples every seven days until day 28 (day 0, day 7, day 14, day 21, and day 28 of the differentiation). Each time point represents a different stage of hepatic differentiation. Days 0 and 7 represent the stages of undifferentiated iPSCs and DE-like cells, respectively. DE marker expression is shown in Fig. 1B. Day 14 is the endpoint of the cultivation with the Progenitor medium, an intermediate stage between DE-like cells and hepatocyte-like cells. Day 21 and Day 28 correspond to the stage of the hepatocyte-like cell. The characteristics of the hepatocyte-like cells were shown in our previous report^10^. Methylome data were acquired using Infinium MethylationEPIC beadchips, and transcriptome data were acquired by CAGE. The DE-like cell differentiation was performed using the Cellartis® Definitive Endoderm Differentiation Kit (Takara Bio Inc.), equivalent to the DE-like cell differentiation step of the Cellartis® Hepatocyte Differentiation Kit (Takara Bio Inc.). The DE-like cell differentiation time-course samples were obtained starting iPS cells and every 6 hours from 48 hours to 72 hours (0, 48, 54, 60, 66, and 72 hours) to cover the timing of GATA6 upregulation. Methylome data acquired by Infinium MethylationEPIC beadchips, transcriptome data acquired by CAGE, GATA6 binding profile acquired by chromatin immunoprecipitation, and chromatin accessibility acquired by OmniATAC-seq were obtained.

### iPS cell culture condition

The 201B7 human iPS cell line, which was derived from the skin of a 36-year-old female with retroviral vectors of the four Yamanaka factors (Oct3/4, Sox2, Klf4, c-Myc), was acquired from the RIKEN BioResource Center (BRC). The iPS cells cultured with mouse STO feeder cells were converted to feeder-free culture conditions using the Cellartis® DEF-CS™ Culture System (Takara Bio Inc.).

### Definitive endoderm-like cell and Hepatocyte-like cell differentiations

iPS cells were sub-cultured at a density of 4.2 × 10^4^ cells/cm^2^ three days before the *in vitro* differentiation. iPSCs were differentiated into DE-like cells using the Cellartis® Definitive Endoderm Differentiation Kit (Takara Bio Inc.) for seven days according to the manufacturer’s instructions. The obtained DE cells were detached and dissociated using TrypLE Select (Thermo Fisher Scientific Inc., Waltham, MA). The DE cells were seeded at 1.3 × 10^5^ cells/cm^2^ and were differentiated into hepatocyte-like cells using Cellartis® Hepatocyte Differentiation Kit (Takara Bio Inc.).

### Immunocytochemistry

The Day 7 DE-like cells cultured on a cover glass were fixed in 4% formaldehyde for 10 min, followed by blocking with 1% skim milk. The cells were incubated for 24 hours at 4 °C with anti-human FOXA2(Abcam, Cambridge, UK; cat no. ab60721, lot no. GR3426450-2) and anti-human SOX17(Abcam; cat no. ab224637, lot no. GR3449941-5) antibodies diluted to 1:00 by the antibody reaction buffer (1% BSA 0.2% Triton-X100 containing D-PBS(+/+)) for 24 hours at 4 °C. After washing in D-PBS(+/+) twice, the cells were incubated for 1 hour at RT with Alexa Fluor 488-conjugated anti-mouse IgG (Thermo Fisher Scientific Inc., Waltham, MA, USA) and Alexa Fluor 594-conjugated anti-rabbit IgG(Thermo Fisher Scientific Inc.) secondary antibodies diluted to 1:0000 by the antibody reaction buffer. The cells were mounted in slow-fade (Thermo Fisher Scientific Inc.) and analyzed by a BZ-X810 fluorescent microscope (Keyence Corporation, Osaka, Japan).

### DNA extraction

Cells were detached using TrypLE Select (Thermo Fisher Scientific Inc.) and pelletized. The cells were stored at −80 °C until use. DNA extraction was performed using NucleoSpin Tissue (Takara Bio Inc.) according to the manufacturer’s instructions.

### Methylation array

Bisulfite conversion was performed using the EZ DNA Methylation-Gold Kit (Zymo Research, Irvine, CA, USA) with 500 μg of genomic DNA. Bisulfite-converted DNA was hybridized to Infinium MethylationEPIC BeadChips according to the manufacturer’s instructions.

### RNA extraction

Cells were detached using TrypLE Select (Thermo Fisher Scientific Inc.) and lysed in 350 μL lysis buffer (LBP) of NecleoSpin RNA Plus (Takara Bio Inc.). The lysed cells were stored at −80 °C until use. RNA extraction was performed using NucleoSpin RNA plus (Takara Bio Inc.) according to the manufacturer’s instructions.

### Cap analysis gene expression

For CAGE library construction, 3 μg of extracted total RNA was reverse-transcribed using SuperScript III reverse transcriptase (Thermo Fisher Scientific Inc.), and a diol residue of the cap structure was biotinylated, followed by RNase I treatment using RNase ONE ribonuclease (Promega Corporation, Madison, WI, USA). The RNA-cDNA hybrids were captured using streptavidin-coated magnetic beads (Thermo Fisher Scientific Inc.), and only single-stranded cDNAs were released from the beads. Barcoded 5′- and 3′- linkers were ligated to the single-stranded cDNA, followed by 2^nd^ strand synthesis using Deep Vent (exo-) DNA polymerase (New England BioLabs, Ipswich, MA, USA). CAGE library construction was performed using three biological replicates. Eight hepatocyte differentiation time-course or seven DE differentiation libraries were equally multiplexed. A multiplexed library was sequenced in a 50 bp single-end on one lane of the Hiseq. 2500 (Illumina Inc., San Diego, CA, USA).

### OmniATAC-sequencing

Cells were detached using TrypLE Select (Thermo Fisher Scientific Inc.) and stored at −80 °C in STEM-CELLBANKER GMP grade (Takara Bio Inc.) until use. The cells were quickly defrozen in a 37 °C water bath. The 5 × 10^4^ cells were washed in PBS twice, and nuclei were extracted in l cold ATAC-Resuspension Buffer (RSB) containing 0.1% NP40, 0.1% Tween20, and 0.01% Digitonin. The nuclei were resuspended in a transposition mixture (25 ul 2x TD buffer, 2.5 ul transposase (100 nM final), 16.5 ul PBS, 0.5 ul 1% digitonin, 0.5 ul 10% Tween-20, 5 ul H2O) and incubate at 37 °C for 30 min in a thermomixer with 1,000 RPM mixing. DNA was extracted from the reaction using the Zymo DNA Clean and Concentrator Kit (Zymo Research, Irvine, CA, USA). The sequencing library was generated using NEBNext Ultra DNA Library Prep Kit for Illumina (New England BioLabs) with five cycles followed by three to seven cycles of pre- and PCR-amplification, respectively. The amplified library was purified with Zymo DNA Clean and Concentrator kit (Zymo Research), followed by size-selection with SPRIselect (1:0.6 and 1:0.2 sample vol. to beads vol.; Beckman Coulter, CA, USA). The fragment size of the OmniATA-seq libraries was checked by Bioanalyzer (Agilent Technologies, Inc., Santa Clara, CA, USA). The fragment size of each library was mainly between 200 bp and 600 bp. OmniATAC was performed in two biological replicates. The concentration of the OmniATAC libraries was measured using KAPALibraryQuantificationKits (F. Hoffmann-La Roche, Ltd., Basel, Swiss Confederation). All the OmniATAC libraries were equally multiplexed and sequenced in a 50 bp single end on one lane of the Hiseq. 2500 (Illumina Inc.).

### GATA6 ChIPmentation sequencing

Cells were detached using TrypLE Select (Thermo Fisher Scientific Inc.) and fixed in 1% formaldehyde for 8 min at RT. The fixation was quenched by adding glycine solution at a final concentration of 200 mM and incubated for 10 min at RT. The fixed cells were snap-frozen in liquid nitrogen and stored at −80 °C until use. ChIP was performed using auto ChIPmentation for TF kit (Diagenode SA., Seraing, Belgium) according to the manufacturer’s instructions. Briefly, two million fixed cells were lysed and sonicated using Picoruptor® (Diagenode SA.) in 1.5 mL Bioruptor Microtubes for ten cycles (1 cycle: 30 s sonication and 30 s “off”) at 4 °C. Magnetic immunoprecipitation and tagmentation were performed on the SX-8G IP-STAR® Compact Automated System (Diagenode SA.) with an anti-GATA6 antibody (D61E4, Cell Signaling Technology, Inc.). Chromatin was extracted from the magnetic beads in the stripping reagent for 30 min at 50 °C, followed by end-repair and reverse cross-linking. Illumina sequencer convertible libraries were generated and amplified by nine cycles of PCR. The optimal size of the sequencing libraries (200 bp) was purified using AMPure XP beads (1:1.8 sample vol. to beads vol.; Beckman Coulter), and the purification result was confirmed by Bioanalyzer (Agilent Technologies, Inc., Santa Clara, CA, USA). ChIPmentation was performed in two biological replicates. The concentration of the ChIPmentation libraries was measured using KAPALibraryQuantificationKits (F. Hoffmann-La Roche, Ltd). All the ChIPmentation libraries were equally mixed and sequenced using 150 bp paired-end reads on one lane of the HiSeq X (Illumina Inc.).

### Computational methods

#### Methylation array data processing

Raw intensity data (IDAT) of Infinium MethylationEPIC BeadChips were read into MethyLumiSet objects using *the readEPIC* function implemented in the watermelon package (version 1.34.0) of R. Color balance adjustment and normalization by quantile normalization were performed using *lumiMethyC* and *lumiMethyN* functions of the lumi package (version 2.42) of R.

#### CAGE data processing

**Fig. 2:**
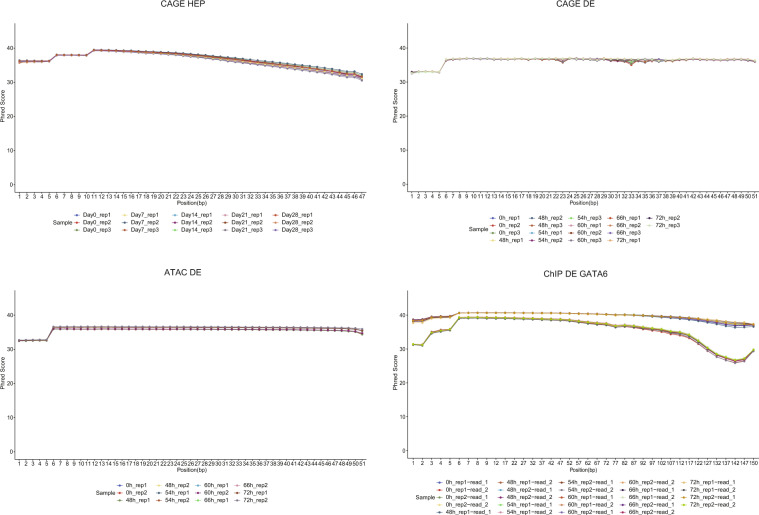
Sequencing data quality. X and Y axis donate the position of sequence and Phred quality score.

Raw sequencing data quality was checked by fastQC, and the results were summarized using MultiQC (Fig. 2). Raw CAGE sequence data were processed using MOIRAI, a web-based CAGE data processing workflow^11^. Briefly, the sequences were trimmed using a fastx-trimmer and fastx_clipper FASTX-toolkit (version 0.0.13). Ribosomal RNA reads were removed using rRNAdust (version 1.02) *(*fantom.gsc.riken.jp/5/suppl/*rRNAdust*/). Then, the sequences were mapped to the hg19 genome using STAR (version 2.5.3a). Mapped CAGE tags were counted for each FANTOM5 promoter (https://fantom.gsc.riken.jp/5/datafiles/latest/extra/CAGE_peaks/hg19.cage_peak_phase1and2combined_ann.txt.gz) using bedtools (version 2.26.0) and normalized as tags per million reads (TPM). The number of reads and mapping rate of each sample are shown in Table 1.

**Table 1:** General statistics of sequencing data.

Analysis	Sample	Error rate	Read Mapped	Mapping rate (%)	% MapQ 0 Reads	Total sequence
CAGE_HEP	day0_rep1	1.83%	9,361,967	100.00	1.72	9,361,967
CAGE_HEP	day0_rep2	1.92%	9,866,368	100.00	1.47	9,866,368
CAGE_HEP	day0_rep3	1.89%	12,502,457	100.00	1.63	12,502,457
CAGE_HEP	day7_rep1	1.93%	13,104,518	100.00	0.70	13,104,518
CAGE_HEP	day7_rep2	1.86%	17,020,928	100.00	0.86	17,020,928
CAGE_HEP	day7_rep3	1.91%	9,882,473	100.00	0.81	9,882,473
CAGE_HEP	day14_rep1	1.97%	12,155,537	100.00	0.42	12,155,537
CAGE_HEP	day14_rep2	1.96%	13,217,780	100.00	0.49	13,217,780
CAGE_HEP	day14_rep3	1.88%	15,377,991	100.00	0.56	15,377,991
CAGE_HEP	day21_rep1	1.99%	10,932,040	100.00	0.38	10,932,040
CAGE_HEP	day21_rep2	1.94%	9,724,140	100.00	0.42	9,724,140
CAGE_HEP	day21_rep3	1.96%	8,216,205	100.00	0.37	8,216,205
CAGE_HEP	day28_rep1	1.95%	5,635,658	100.00	0.41	5,635,658
CAGE_HEP	day28_rep2	2.01%	6,632,429	100.00	0.36	6,632,429
CAGE_HEP	day28_rep3	1.99%	4,919,875	100.00	0.35	4,919,875
CAGE_DE	0h_rep1	0.00%	15,787,242	100.00	1.06	15,787,242
CAGE_DE	0h_rep2	0.00%	14,557,688	100.00	1.10	14,557,688
CAGE_DE	0h_rep3	0.00%	14,961,744	100.00	1.07	14,961,744
CAGE_DE	48h_rep1	0.00%	16,902,027	100.00	1.00	16,902,027
CAGE_DE	48h_rep2	0.00%	16,676,173	100.00	1.03	16,676,173
CAGE_DE	48h_rep3	0.00%	16,052,742	100.00	1.05	16,052,742
CAGE_DE	54h_rep1	0.00%	15,722,626	100.00	1.04	15,722,626
CAGE_DE	54h_rep2	0.00%	14,291,113	100.00	1.06	14,291,113
CAGE_DE	54h_rep3	0.00%	14,572,886	100.00	1.02	14,572,886
CAGE_DE	60h_rep1	0.00%	15,927,485	100.00	0.99	15,927,485
CAGE_DE	60h_rep2	0.00%	13,697,716	100.00	1.09	13,697,716
CAGE_DE	60h_rep3	0.00%	14,805,159	100.00	1.04	14,805,159
CAGE_DE	66h_rep1	0.00%	15,086,488	100.00	1.08	15,086,488
CAGE_DE	66h_rep2	0.00%	14,030,817	100.00	1.03	14,030,817
CAGE_DE	66h_rep3	0.00%	14,649,200	100.00	1.03	14,649,200
CAGE_DE	72h_rep1	0.00%	16,797,309	100.00	1.01	16,797,309
CAGE_DE	72h_rep2	0.00%	15,463,863	100.00	1.03	15,463,863
CAGE_DE	72h_rep3	0.00%	15,535,466	100.00	1.00	15,535,466
ATAC_DE	0h_rep1	0.26%	31,269,727	99.35	0.54	31,474,851
ATAC_DE	0h_rep2	0.32%	25,285,725	98.97	0.87	25,548,574
ATAC_DE	48h_rep1	0.35%	22,216,006	98.90	0.98	22,463,192
ATAC_DE	48h_rep2	0.30%	20,278,584	98.97	0.85	20,489,896
ATAC_DE	54h_rep1	0.29%	24,951,051	99.15	0.75	25,165,253
ATAC_DE	54h_rep2	0.32%	22,929,382	98.81	1.04	23,205,281
ATAC_DE	60h_rep1	0.31%	36,035,569	98.81	0.95	36,468,062
ATAC_DE	60h_rep2	0.34%	23,431,573	98.75	1.03	23,729,220
ATAC_DE	66h_rep1	0.29%	30,388,066	98.94	0.82	30,714,608
ATAC_DE	66h_rep2	0.36%	24,137,917	98.34	1.33	24,544,713
ATAC_DE	72h_rep1	0.38%	21,218,162	98.28	1.37	21,588,568
ATAC_DE	72h_rep2	0.37%	24,475,708	98.30	1.35	24,899,095
ChIP_DE_GATA6	0h_rep1	0.63%	27,032,116	95.66	1.63	28,257,068
ChIP_DE_GATA6	0h_rep2	0.60%	19,004,429	95.99	1.62	19,798,676
ChIP_DE_GATA6	48h_rep1	0.54%	28,189,167	96.38	1.26	29,249,332
ChIP_DE_GATA6	48h_rep2	0.57%	11,478,791	96.59	1.24	11,883,728
ChIP_DE_GATA6	54h_rep1	0.53%	79,940,080	96.92	1.17	82,482,222
ChIP_DE_GATA6	54h_rep2	0.54%	57,816,543	96.87	1.26	59,687,516
ChIP_DE_GATA6	60h_rep1	0.59%	9,958,979	96.55	1.25	10,314,592
ChIP_DE_GATA6	60h_rep2	0.55%	66,828,073	96.87	1.22	68,987,538
ChIP_DE_GATA6	66h_rep1	0.61%	84,871,087	96.36	1.32	88,081,440
ChIP_DE_GATA6	66h_rep2	0.54%	41,684,312	96.74	1.24	43,088,884
ChIP_DE_GATA6	72h_rep1	0.68%	69,525,626	96.50	1.20	72,048,654
ChIP_DE_GATA6	72h_rep2	0.58%	83,416,231	96.59	1.35	86,358,204

#### OmniATAC-seq data processing

Raw sequencing data quality was checked by fastQC, and the results were summarized using MultiQC (Fig. 2). Sequence reads were mapped to the hg19 genome using bowtie2 (version 2.3.0). The mapped reads were frozen, and reads mapped to the mitochondrial genome were removed using the removeChrom.py script of the Harvard ATAC-seq module. Peak calling was performed using MACS2 (version 2.1.1.20160309) with the following parameters:--shift −37,–extsize 73, -B –SPMR, and -p 10e-6. Peaks that overlapped with the ENCODE blacklist regions were removed. Bigwig coverage files were generated using the bam2wig.py script (version 2.6.4) of RSeQC with wigToBigWig (version 2.8). The number of reads and mapping rate of each sample are shown in Table 1.

#### ChIPmentation sequence data processing

Raw sequencing data quality was checked by fastQC, and the results were summarized using MultiQC (Fig. 2). Sequence reads were mapped to the hg19 genome using bowtie2(version 2.3.0) and reads mapped to the mitochondrial genome, and PCR duplicates were removed using the removeChrom.py script of the Harvard ATAC-seq module and samtools (version 1.9). Peak calling was performed using MACS2 (version 2.1.1.20160309) with a cut-off p-value < 10^−6^, and peaks that overlapped with the ENCODE blacklist regions were removed. Bigwig coverage files were generated, as described above. The number of reads and mapping rate of each sample are shown in Table 1.

## Data Records

The raw fastq files, CTSS bed files and expression tables of CAGE, bigwig coverage files of omniATAC-seq, bigwig coverage files, and peak summit bed files were deposited at the Gene Expression Omnibus (https://www.ncbi.nlm.nih.gov/geo/) under the accession ID of SuperSeries GSE163331^12^. The SuperSeries comprised the methylation array data SubSeries for hepatocyte-like cell differentiation (GSE163324) and DE-like cell differentiation (GSE163322), CAGE data SubSeries for hepatocyte-like cell differentiation (GSE163329), DE-like cell differentiation (GSE163328), omniATAC-seq data SubSeries for DE-like cell differentiation (GSE163327), and GATA6 chromatin immunoprecipitation data for DE-like cell differentiation (GSE163330).

## Technical Validation

### Validation of CAGE reproducibility

**Fig. 3:**
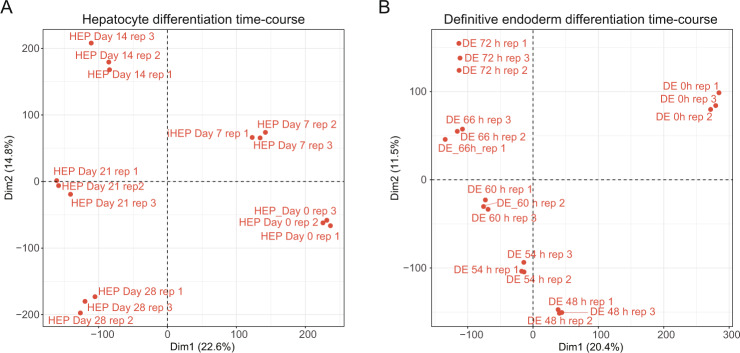
Two-dimensional projection of PCA for each CAGE data of hepatocyte differentiation (**A**) and definitive endoderm differentiation (**B**). X and Y axes are principal component 1 (Dim 1) and principal component 2 (Dim 2). The percentage written in the bracket at each dimension represents eigen value.

To evaluate the reproducibility of CAGE, we performed a principal component analysis based on the expression table (Fig. 3A,B). The replicate data for each time point were closely plotted in the 2D space, indicating high reproducibility.

### *De novo* motif analysis of GATA6 ChIPmentation

**Fig. 4:**
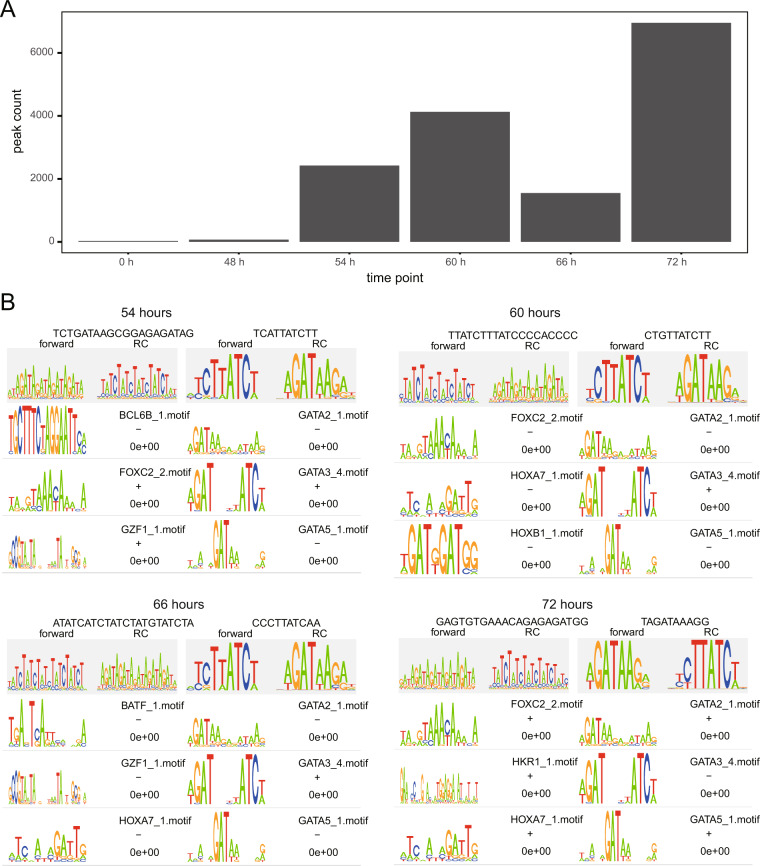
Technical validation of GATA6 ChIPmentation data. (**A**) A bar plot represents the peak count of each time point. The peaks were identified using MACS2 with a p-value < 10^−6^. (**B**) Motif overrepresented at ChIPpeaks. The top tiers with a gray background are the overrepresented *de novo* motifs. The lower tiers are known transcription factor-binding motifs similar to the overrepresented *de novo* motifs.

To examine the enriched sequences by GATA6 ChIPmentation, *de novo* motif analysis was performed. Because there were few peaks at 0 h and 48 h (Fig. 4A), we analyzed time points of 56, 60, 66, and 72 h. At all analyzed time points, we detected GATA sequences containing motifs that were significantly similar to the known GATA motifs (Fig. 4B).

### Read coverage distribution of OmniATAC-seq

**Fig. 5:**
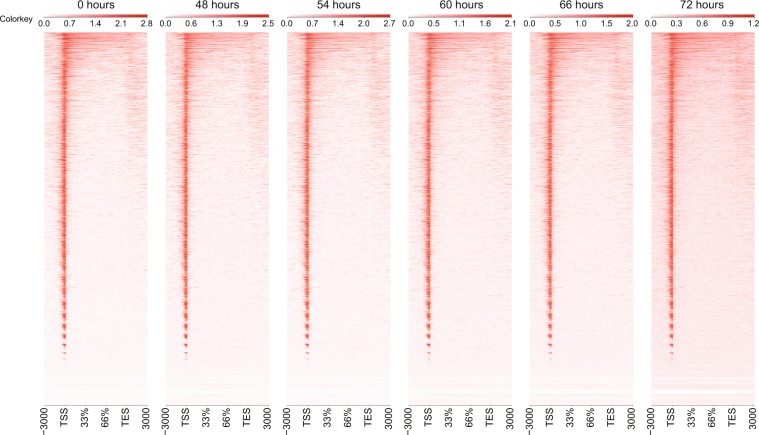
OmniATAC-seq reads distribution around genes. The column shows the region of 3000 bp upstream from the transcription start site (TSS) to 3000 bp downstream from the transcription end site (TES). Rows are genes. The enrichment of reads is shown as a gradient of red.

Highly accessible regions are enriched in gene regulatory regions, such as promoters^13^. Therefore, we analyzed the distribution of OmniATAC-seq reads around known gene models. The OmniATAC-seq reads were highly enriched at transcription start sites (TSSs) (Fig. 5).

### Methylation-level distribution of methylation array

**Fig. 6:**
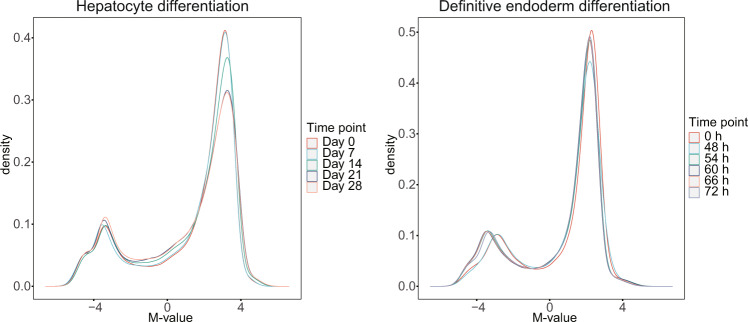
Distributions of M-values in hepatocyte differentiation time-course data (left) and definitive endoderm differentiation time-course data (Right).

The M-value of methylation assay data typically shows a bimodal distribution, representing hypomethylated and hypermethylated CpGs^14^. Our methylation array M-values also showed bimodal distribution (Fig. 6).

## Data Availability

The code for the data pre-processing and technical validations are available on GitHub (https://github.com/RIKEN-CFCT/hep_methyl_data).
